# Triboelectric nanogenerators as wearable power sources and self-powered sensors

**DOI:** 10.1093/nsr/nwac170

**Published:** 2022-08-29

**Authors:** Xiong Pu, Chi Zhang, Zhong Lin Wang

**Affiliations:** CAS Center for Excellence in Nanoscience, Beijing Key Laboratory of Micro-Nano Energy and Sensor, Beijing Institute of Nanoenergy and Nanosystems, Chinese Academy of Sciences, Beijing 101400, China; School of Nanoscience and Technology, University of Chinese Academy of Sciences, Beijing 100049, China; CAS Center for Excellence in Nanoscience, Beijing Key Laboratory of Micro-Nano Energy and Sensor, Beijing Institute of Nanoenergy and Nanosystems, Chinese Academy of Sciences, Beijing 101400, China; School of Nanoscience and Technology, University of Chinese Academy of Sciences, Beijing 100049, China; CAS Center for Excellence in Nanoscience, Beijing Key Laboratory of Micro-Nano Energy and Sensor, Beijing Institute of Nanoenergy and Nanosystems, Chinese Academy of Sciences, Beijing 101400, China; School of Nanoscience and Technology, University of Chinese Academy of Sciences, Beijing 100049, China; School of Materials Science and Engineering, College of Engineering, Georgia Institute of Technology, Atlanta, GA 30332, USA

**Keywords:** triboelectric nanogenerators, power sources, self-powered sensors, wearable electronics

## Abstract

Smart wearable technologies are augmenting human bodies beyond our biological capabilities in communication, healthcare and recreation. Energy supply and information acquisition are essential for wearable electronics, whereas the increasing demands in multifunction are raising the requirements for energy and sensor devices. The triboelectric nanogenerator (TENG), proven to be able to convert various mechanical energies into electricity, can fulfill either of these two functions and therefore has drawn extensive attention and research efforts worldwide. The everyday life of a human body produces considerable mechanical energies and, in the meantime, the human body communicates mainly through mechanical signals, such as sound, body gestures and muscle movements. Therefore, the TENG has been intensively studied to serve as either wearable sources or wearable self-powered sensors. Herein, the recent finding on the fundamental understanding of TENGs is revisited briefly, followed by a summary of recent advancements in TENG-based wearable power sources and self-powered sensors. The challenges and prospects of this area are given as well.

## Teaser

Research progresses of the triboelectric nanogenerator as a promising mechanical-to-electrical energy conversion technology are comprehensively reviewed for application in wearable power sources and self-powered sensors.

## INTRODUCTION

Powering miniature wearable electronics has long been a challenge. State-of-the-art wearable electronics rely on the energy supply from electrochemical energy-storage devices, especially lithium-ion batteries [1]. Nevertheless, the increasing functionalities and intelligence of the wearable electronics have led to growing demands for higher energy but smaller weight and/or volume. Therefore, tremendous efforts have been made, on the one hand, to develop wearable energy-harvesting devices as sustainable power sources and, on the other, to design low-power-consumption electronics or self-powered sensors [2]. Strategies have been proposed to harvest thermal, mechanical and chemical energies of human bodies or nearby environments [3], among which mechanical energies could play crucial roles in wearable electronics from the following two aspects. First, the everyday life of a human body produces considerable mechanical energies, which could provide a significant amount of energy for wearable electronics if harvested efficiently; second, the human body communicates mainly through mechanical signals, such as sound, body gestures and muscle movements, which could be utilized for sensors or human-interfaced electronics with sensitive mechanical-to-electrical converters.

The triboelectric nanogenerator (TENG) has drawn extensive attention since first reported in 2012 [4]. By coupling the effects of contact electrification and electrostatic induction, the TENG can convert various types of mechanical energies into electricity, including body motions, water waves, winds, vibrations and so on [5]. Due to the following two aspects, it is demonstrated to be highly promising for wearable electronics. First, the TENG shows great performances for mechanical energies at low frequency (<5 Hz), which is suitable for harvesting most human body-related motion energies [6]. Second, the structures and materials can be facilely designed in order to adapt to different wearable applications. As the contact electrification is a universal effect, the TENG can be realized with dielectric, semiconducting and electrolytic materials in solid, liquid or even gas phases [7]. These materials could be processed into either 1D fibers/yarns, 2D films/fabrics or 3D architectures [8]. Furthermore, the TENG has been reported to be designed into stretchable, biodegradable, bioabsorbable or implantable devices, fulfilling a variety of multifunctional wearable applications [9].

**Figure 1.**
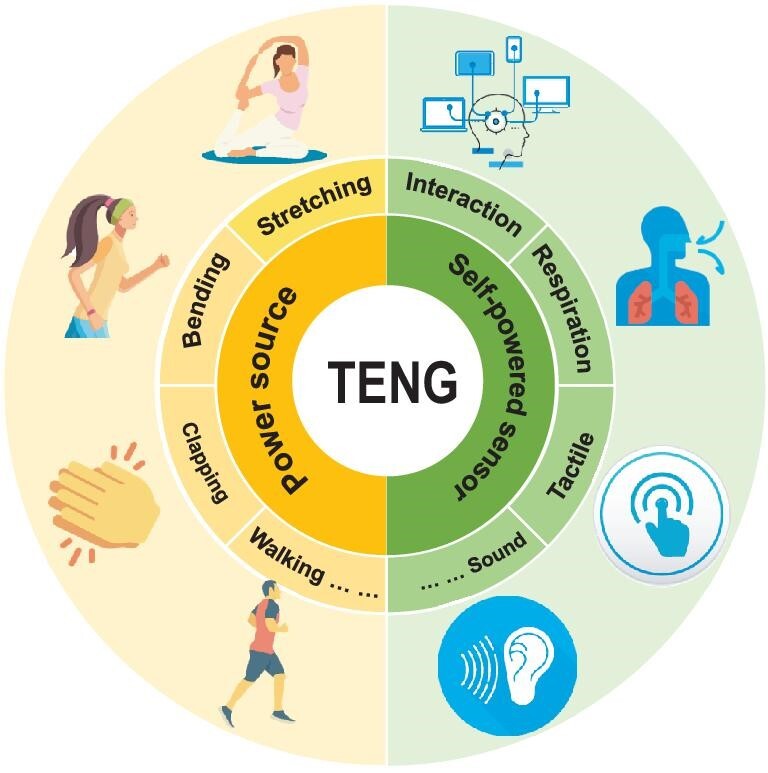
The scheme of TENGs as wearable power sources and self-powered sensors for wearable electronics.

Despite the existing review papers on TENGs from different aspects [10,11], a comprehensive summary of the state-of-the-art developments of TENGs as power sources and self-powered sensors specifically for wearable electronics is necessary, as shown in Fig. 1. A brief summary of the fundamental origins of the TENGs will be given first, including recent findings on the mechanisms of the electrification process and electricity-generation process. The materials engineering for TENGs will be then discussed. In addition, representative progress of TENGs as wearable power sources and self-powered sensors will be elaborated on, followed by provision of the prospects of the challenges and opportunities in this field.

## FUNDAMENTALS OF THE TENG

Fundamental studies of the TENG are crucial for improving the performance and exploring innovative applications. A simple physical picture of the TENG contains typically two steps: the generation of electrostatic charges through the contact electrification effect and the generation of external electrical current through the electrostatic induction effect. Nevertheless, the comprehensively theoretical description could be much more complex. Recent efforts have achieved in-depth understandings on the theoretical origins of both of the two processes.

**Figure 2.**
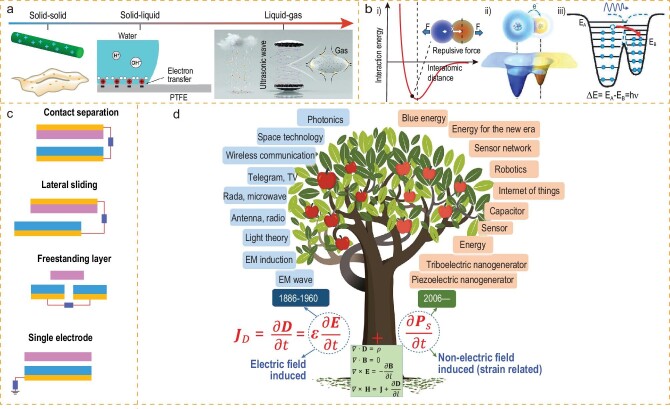
The fundamentals of a TENG. (a) The interface between different states of matter, solid–solid, solid–liquid [112], liquid–gas [18]. Copyright 2019, WILEY-VCH Verlag GmbH & Co. KGaA, Weinheim; Copyright 2019, The Royal Society of Chemistry. (b) Schematic diagram of electron cloud and potential energy trap model [7]. Copyright 2020, WILEY-VCH Verlag GmbH & Co. KGaA, Weinheim. (c) Four modes of motion for a TENG [23]. (d) Theoretical basis for TENGs with a tree model illustrating that the newly added second term $\frac{{\partial {P}_s}}{{\partial t}}$ of Maxwell's displacement current corresponds to the theoretical basis for TENGs in sensing and energy-harvesting applications. Reproduced with permission [24]. Copyright 2019, Elsevier.

Contact electrification is a phenomenon that has been documented for >2600 years, but its scientific understanding is still incomprehensive and inconclusive. As this effect exists in nearly all materials in either solid, liquid or gas phases, no unified physical description or model has been proposed and accepted before (Fig. 2a). There are disputes arguing that the surface is charged through the transfer of ions, electrons or material species at the interfaces. However, the systematic investigations reported recently support that electron transfer is the dominant mechanism for the electrification process at either the solid, liquid or gas interfaces [12,13]. Representative experiments strongly supporting this conclusion include the following examples. At metal–dielectric interfaces, Kelvin probe force microscopy (KPFM) study showed that the polarity of the charges generated on the dielectric surfaces can be tuned by the bias voltage applied to the metal tip [14]. As the effective Fermi level of the metal can be tuned to be higher or lower than the empty surface states of the dielectrics, the dielectric surface will accept electrons from or donate electrons to the metal, respectively. The KPFM study also showed that the contact electrification of two materials at different temperatures coincides with the electron-transfer mechanism [15]. Other strong evidence is that the obtained charges trapped in the surface states of dielectric materials dissipate at high temperatures according to the electron thermionic emission principle [16]. At liquid–solid interfaces, the tribocharges generated by sliding a water droplet on a dielectric surface were dominantly contributed by electron transfer because the majority of the charges also dissipated through electron thermionic emission and only a small portion of residual ‘sticky’ charges were attributed to ion adsorption [17]. This finding could potentially lead to new understandings on the formation of electric double layers at liquid–solid interfaces and a two-step model was proposed. Even at the liquid–gas interface, electrification made the water droplet positively charged [18]. A very recent study even showed that photoemission was detected during the contact electrification process between two dielectric surfaces [19]. This result not only strongly supports that electron transfer dominates the electrification, but also suggests exciting new techniques such as contact electrification-induced interface spectroscopy since photo emission carries finger-print information of electron energies of interface atoms. Furthermore, study of the electrification mechanism leads to a new promising catalysis approach that electron exchanges at the water–dielectric interface induce the degradation of methyl orange aqueous solution [20]. Therefore, this progress not only clarifies the contact electrification mechanism but also suggests promising new technologies.

A general contact electrification model was then proposed based on this experimental evidence (Fig. 2b). For many materials with well-defined molecular structures or electronic structures, a band diagram can be simply applied to explain the electrification. Nevertheless, there are also a large number of materials that are hard to describe using a band diagram. A simplified model using an interatomic interaction potential model can be adopted to explain the electrification process [12,16]. When two surfaces make contact, the atoms of the two materials have to be close enough to start the electron transfer, and the distance has to be in the repulsive region of the potential well. This has been well supported by KPFM studies [21] and quantum mechanics modeling [22]. After being separated, the transferred electrons will be trapped in the surface or defect states as static charges. Furthermore, this electron-transfer process can excite photo emissions through possible processes as detailed in Wang *et al.*’s report [19].

Four typical modes of TENGs are designed to convert mechanical motion energy into electricity, i.e. the vertical contact-separation mode, lateral sliding mode, single-electrode mode and free-standing mode (Fig. 2c) [23]. The basic theoretical origin of the electricity-generation process lies in the Maxwell's displacement current, which is not the conduction current resulting from the flux of moving free charges but is due to the time-varying electric field [24–26]. The contact electrification process results in the charged surfaces with electrostatic charges being immobile in the dielectric media to produce conduction current. Nevertheless, the mechanical motion of the charged surface will make the electric field of these static charges time-varying. This displacement current is different from the medium polarization (*P*) induced by an applied electric field (*E*), but it is related to the polarization (*P_s_*) of the surface electrostatic charges. The *P* is dependent on the applied electric field but the *P_s_* is mechanical-motion-driven. Therefore, to clearly show its physical meaning and to calculate the electrical outputs of the TENG, Wang added the polarization term *P_s_* in the displacement vector and the Maxwell's displacement current (*J_D_*) can be rewritten as [24]:

(1) \begin{equation*} {\rm{\ }}{{\boldsymbol{J}}}_{\boldsymbol{\!D}} = \frac{{\partial {\boldsymbol{D}}}}{{\partial t}}\ = \ \varepsilon \frac{{\partial {\boldsymbol{E}}}}{{\partial t}} + \frac{{\partial {{\boldsymbol{P}}}_{\boldsymbol{s}}}}{{\partial t}},\ \end{equation*}

(2) \begin{equation*} {\boldsymbol{D\ }} = \ \varepsilon {\boldsymbol{E}} + {{\boldsymbol{P}}}_{\boldsymbol{s}}, \end{equation*}

where ***D*** is the electric displacement vector, *ϵ* is the dielectric constant and *t* is the time. The term $\frac{{\partial {{\boldsymbol{P}}}_{\boldsymbol{s}}}}{{\partial t}}$ is caused by mechanical motion or external strain field. The main fundamental science and technological applications derived from the Maxwell's displacement current are shown in Fig. 2d.

Recently, for a media system that moves at a time-dependent velocity (e.g. in a non-inertia frame), the Maxwell equations have been expanded by Wang as follows for describing their electromagnetic behavior [26,27]:

(3a) \begin{eqnarray*} \nabla \ \cdot \ {\boldsymbol{D}}^{\prime} = \ {\rho }_{f\ } - \nabla \cdot {\boldsymbol{P}}_s, \end{eqnarray*}

(3b) \begin{eqnarray*} \nabla \cdot {\boldsymbol{B}}\ = \ 0,\ \end{eqnarray*}

(3c) \begin{eqnarray*} \nabla \times \ ({\boldsymbol{E}}\ - \ {\boldsymbol{v}} \times {\boldsymbol{B}}\!)\ = \ - \frac{\partial }{{\partial t}}{\boldsymbol{B}},\ \end{eqnarray*}

(3d) \begin{eqnarray*} &&\nabla \times [{\boldsymbol H} + {\boldsymbol{v}} \times ({\boldsymbol D}^{\prime}{\rm{ + \ }}{\boldsymbol P}_s)] = {\boldsymbol J}_{\!\!\!f}{\rm{ + }}{\rho }_{f }{\boldsymbol{v\ }}\\ &&\quad + \frac{\partial }{{\partial t}}[{\boldsymbol D}^{\prime} + {\boldsymbol P}_s], \end{eqnarray*}

which are referred to as the general Maxwell's equations for mechano-driven, slow-moving media at an arbitrary velocity field. The equations describe the coupling among mechanical, electrical and magnetic properties and behaviors of the system that moves with acceleration. These equations are based on the assumptions that: (i) the velocity is time-varying and far smaller than light speed (*v* ≪ c) and (ii) the relativity effect is approximately ignored. Due to the mechanical motions or deformations, the boundary of the media system is time-varying, e.g. its shape and volume can change at an arbitrary velocity field. That is, these equations are a derived non-inertia frame rather than the uniform motion of the media along a straight line in an inertia frame. As the mechanical energy input couples with electricity and magnetism, it may not satisfy Lorentz covariance. Nevertheless, from an engineering point of view, the equations are applicable to charged media in not only solid/soft form but also in fluid/liquid form.

## MATERIALS FOR THE TENG

The triboelectric effect is ubiquitous, nearly existing between all different materials or even the same material. It means that almost all materials could be used to fabricate TENGs. This section will discuss the materials engineering for TENGs from two aspects, i.e. triboelectrification materials and electrode materials.

For the triboelectrification materials, insulators, semiconductors and conductors could all be selected. Insulators or dielectric materials are commonly used to serve as the triboelectrification layer of the TENG. The insulating polymers are the most utilized triboelectrification materials, as the electrostatic charges could not be easily dissipated on their surfaces. The output voltage and power of a TENG are typically positively proportional to the static charge quantities maintained on the surface of the electrification polymers. The materials engineering is then the core concern to boost the output. The electrification properties of a large number of polymer materials have been studied, trying to quantitatively establish the triboelectric series [28]. Owing to the flexibility of these polymer materials, they are quite suitable for developing flexible, stretchable or wearable TENGs. Intensive attention is paid to enrich the functionalities of the TENG through electrification polymer designs. Many functions can be obtained by appropriately designing the structures of the electrification polymers of a TENG device, such as stretchability, wearability, transparency, self-healing capability, biocompatibility and biodegradability.

Semiconductor materials have recently been found to be utilized as electrification materials for fabricating direct-current (DC) TENGs based on a newly discovered effect, i.e. the tribovoltaic effect [12,29,30]. At the metal–semiconductor interface or semiconductor–semiconductor interface, sliding motion could excite non-equilibrium electron-hole pairs to output direct current in the external circuit, analogous to the photon-excited photovoltaic effect. It is still not clearly proven how the electron-hole pairs are excited, but we propose that the close contact of the two surface atoms leads to the formation of dynamic atom–atom bonds and the released energy contributes to the excitation energy. This effect is found in inorganic semiconductors and organic semiconductors, and the latter are suitable for fabricating flexible or wearable energy harvesters [31].

Conductive materials also could be served as triboelectrification materials. Many metals can readily donate free electrons and are shown to be tribopositive when serving as the electrification material [32]. Liquid metals were demonstrated to be effective probing materials to determine the triboelectric series of different materials [33]. Recently, the ionic conductive hydrogels were shown to be an electrification material [34]. The electrification between a dielectric polymer and the hydrogel could lead to the formation of a mechanical-motion-induced time-varying electric double layer at the interface, which is then attributed to the output of alternating current in external circuits.

A variety of different types of conductive materials have been designed as the electrode materials of TENGs. Among them, metals were widely adopted due to their excellent conductivity and easy availability [35,36]. Liquid metals were reported to be encapsulated into some flexible or stretchable triboelectrification materials to serve as self-adaption electrodes [37]. Composite materials were also constructed by dispersing conductive nanofibers or nanoparticles into the stretchable matrix to obtain both conductivity and stretchability. For example, a stretchable porous TENG was fabricated based on the hybrid structure of multiwall carbon nanotubes and polydimethylsiloxane (PDMS) matrix [38]. This TENG could be stretched to 243%. Other than the electron conductive materials, ionic conductors were also developed for the electrode. With a hydrogel electrode, ultra-high stretchability and high transparency were achieved simultaneously for the TENG [39]. Hydrogel materials also could be fabricated to a fiber and then constructed into a woven-shaped TENG [40]. The woven structure enabled the TENG to have excellent 2D extensibility compared with a single fiber and great permeability compared with film shapes. To solve the evaporation or leakage issue of hydrogels, a dynamically cross-linked dry ion-conducting elastomer was then reported to achieve high stretchability and environmental stability simultaneously [41].

Recently, several biodegradable or even natural materials were also chosen to fabricate TENGs [42]. For biodegradable materials, cellulose, chitin, silk fibroin, egg white and rice paper could be selections. Specifically, a fully biodegradable vertical contact-separation mode TENG has been developed [43]. Egg white and rice paper were selected as the electrification materials and an ultra-thin magnesium film was selected as the electrode. Silk fibroin films were adopted as the encapsulation layer. The max electrical output of this TENG could reach 21.6 mW/m^2^. By adjusting the thickness of the encapsulation layer, the degradation time of the TENG could be controlled from days to weeks. As for natural materials, leaves, petal roses, lotus, etc. could be options. For example, owing to the water and electrolyte contained inside, a Hosta leaf has been selected to successfully prepare a single-electrode-mode TENG [44]. The maximum output power of this TENG could reach ∼45 mW/m^2^, which is sufficient to power some commercial electronics, such as LEDs or temperature sensors.

## TENGS AS WEARABLE POWER SOURCES

**Figure 3.**
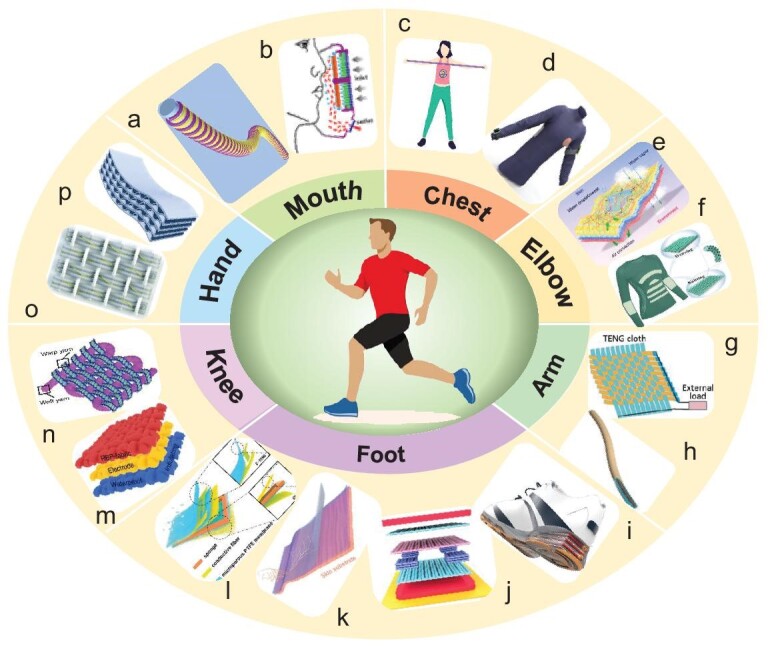
The human body is rich in various forms of mechanical energy and by designing different structures of wearable TENGs with various triboelectric materials to harvest biomechanical energy, they can be used as power sources. (a and b) Mouth [45,46]. Copyright 2022, American Chemical Society; Copyright 2018, American Chemical Society. (c and d) Chest [47,48]. Copyright 2021, American Chemical Society; Copyright 2021, Elsevier. (e and f) Elbow [49,50]. Copyright 2019, Springer Nature; Copyright 2022, WILEY-VCH Verlag GmbH & Co. KGaA, Weinheim. (g and h) Arm [51,52]. Copyright 2015, WILEY-VCH Verlag GmbH & Co. KGaA, Weinheim; Copyright 2018, WILEY-VCH Verlag GmbH & Co. KGaA, Weinheim. (i–l) Foot [53–56]. Copyright 2015, Springer Nature; Copyright 2021, WILEY-VCH Verlag GmbH & Co. KGaA, Weinheim; Copyright 2021, WILEY-VCH Verlag GmbH & Co. KGaA, Weinheim; Copyright 2018, WILEY-VCH Verlag GmbH & Co. KGaA, Weinheim. (m and n) Knee [57,58]. Copyright 2018, Springer Nature; Copyright 2016, Springer Nature. (o and p) Hand [59,60]. Copyright 2020, The Royal Society of Chemistry; Copyright 2016, WILEY-VCH Verlag GmbH & Co. KGaA, Weinheim.

The human body is rich in various forms of mechanical energies, such as hand clapping, knee bending, walking, running, arm swinging, elbow bending, chest stretching and mouth respiration (Fig. 3). As human motions are almost all at low frequency, the TENG could potentially outperform other mechanical energy harvesters due to its better low-frequency response and easier design for flexibility/wearability. Mechanical energies of different parts of human body have been reported to be harvested by wearable TENGs. As for the respiration motion, a real-time smart respiration monitoring system has been fabricated with a comfort fiber-shaped TENG to convert respiration motion into electrical signals, in which the structure design of helical fiber shows high sensitivity to strain. It can automatically send a signal by mobile phone to respond to respiratory behavior changes (Fig. 3a) [45]. By collecting energies of repeated respiratory motion, an electrostatically enhanced wearable respiratory mask has been designed and validated for sustained filtration of ultrafine particles (particle size <1 μm) (Fig. 3b) [46]. The diastolic motion of the chest can also be collected as a wearable energy source. For example, Xu *et al.* and Sheng *et al.* designed a textile-based TENG to collect such energy (Fig. 3c)and d). A hydrogel-based TENG (SH-TENG) with high transparency (>95%) and stretchability (>10 000%) was reported for harvesting typical human movement by Sheng *et al.*, which can concurrently light up 234 LEDs [47]. Xu *et al.* proposed scalable fabrication of a textile TENG by employing a plating stitch technique, which achieves a peak power density of 66.13 mW/m^2^ [48]. As for elbow-motion energy, a TENG can be designed in the shape of an elbow guard or fixed in the bending area of the elbow. Yang *et al.* reported an all-fiber triboferroelectric synergistic e-textile for harvesting this energy and the maximum peak power density is 5.2 W/m^2^ (Fig. 3e) [49]. To achieve large-scale fabrication, Li *et al.* proposed a core–shell triboelectric woven fiber that is further woven into a textile-based TENG, which can be sewn directly into intimate apparel, such as at the elbows and chest. Output electrical energy can light up 116 LEDs simultaneously and can be used as a plantar pressure sensor (Fig. 3f) [50]. Frictional movements between the arm and the axilla can also be collected by a wearable TENG. Pu *et al.* reported a self-charging power unit consisting of a textile-based TENG and lithium-ion battery (LIB) belt, which can continually scavenge the energies of various modes of human motion (Fig. 3g) [51]. Liu *et al.* fabricated a biomechanical energy harvester with a surface charge density of ≤175.4 μC/m^2^ that has the potential to harvest frictional movements between the arm and axilla by taking advantage of the easy encapsulation, wearability and flexibility of tubular TENGs (Fig. 3h) [52]. For daily exercise such as walking or running, Niu *et al.* reported a multi-layer TENG (7.344 W/m^3^)-based universal self-charging system by collecting random biomechanical energy and it realizes a sustainable energy supply to portable electronics with power management (Fig. 3i) [53]. To utilize a multi-material, multi-step 3D printing preparation method, Liu *et al.* fabricated a contact-separation (CS)-mode wearable TENG as a biomechanical energy harvester used to construct a variety of body energy collectors, such as plantar energy collector and an energy-harvesting bracelet (Fig. 3j) [54]. With the aim of improving energy conversion efficiency or achieving self-powered multiple functions, Bai *et al.* and Yuan *et al.* designed solid-state wearable TENGs with different structures to achieve energy harvesting such as human walking or running, respectively. Power densities of 2.3 and 1.03 W/m^2^ were reached, respectively (Fig. 3k)and l) [55,56]. When humans are walking or running, the knees are bending periodically. Researchers have focused their targets for mechanical energy harvesting on these locations in the human body as well. Xiong *et al.* and Gong *et al.* have designed textile-based wearable TENGs with different structures and these energy harvesters have been optimized in terms of collection efficiency in addition to breathability, flexibility, stretchability and washability (Fig. 3m)and n) [57,58]. The desired high output (∼880 V, ∼1.1 μA/cm^2^) and 12.5 μW/m can be obtained, respectively. Hand clapping is one of the most common human movements and several research efforts have demonstrated the utilization of TENGs to collect this human movement. Chen *et al.* designed a negative Poisson's ratio yarn with a composite structure and fabricated some energy-harvesting devices by using a high-speed ring-spinning method (Fig. 3o) [59]. Li *et al.* focused on multi-layer elastomeric TENGs with an outstanding volume charge density of ∼0.055 C/m^3^ as a sustainable energy source to supply electrical energy to wearable electronics (Fig. 3p) [60].

Intensive efforts have further been made to improve the power output of wearable TENGs. TENG output performance is positively proportional to the surface charge density and this criterion is also applicable to wearable TENGs. It is clear that the surface charge density is limited to the minimum of the following three factors: generated static charge density, air breakdown charge density and dielectric breakdown charge density. The first approach is to increase the generated static charges [61,62]. There are many effective methods demonstrated to enhance surface charges. Initially, efforts were made to improve the charge density by increasing the effective surface areas or introducing surface functional groups [63]. The former can be achieved by designing microstructures on the surface, which increase the contact areas at deformed states; the latter was implemented to tune the tribonegativity of the surfaces. Recently, progress has been made in realizing charge-injection methods, such as corona charging [64], charge pumping [65] and charge-excitation and self-polarization of polar high-k materials [62]. An ultra-high charge density of 3.53 mC/m^2^ was acquired by Wu *et al.* in 2022, which paved a new way for further improving the wearable TENG [66]. The second approach is to increase the maximum air breakdown charge density by reliable encapsulation or the addition of an inert gas. Han *et al.* systematically investigated the effects of environmental gas content on TENG output performance [67]. Zi *et al.* also found that high-voltage gas SF_6_ could prevent air breakdown and improve the charge density [68]. Dielectric breakdown is typically very high and it is not a worry with most TENGs. Nevertheless, it is proven that improving the dielectric constant of the electrification layers can boost TENG outputs. Some more detailed approaches for improving high-performance TENGs can be found in a recent review paper [62].

**Figure 4.**
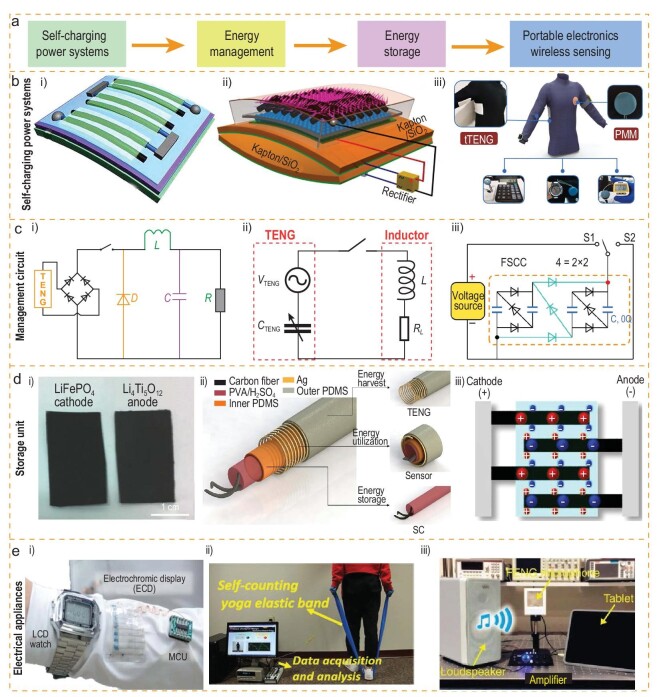
TENG as a self-charging power system (SCPS) consisting of four components: energy-harvesting device, energy-management circuit, energy-storage unit and electrical appliances. (a) Four components of an SCPS. (b) Textile-based SCPS: (i) SCPS with solid supercapacitor as storage unit [69]. Copyright 2012, American Chemical Society; (ii) textile-based supercapacitor charged by motion [70]. Copyright 2013, American Chemical Society; (iii) SCPS consisting of textile TENG and power-management module [48]. Copyright 2018, Elsevier. (c) Three power-management circuits for a TENG: (i) buck chopper circuit for maximum extraction of electrical energy from a TENG [72]. Copyright 2017, Elsevier; (ii) fractal-design-based switched-capacitor convertors for improving energy conversion, lowering the output impedance [75]. Copyright 2020, Springer Nature; (iii) by leveraging the management strength of the opposite-charge enhancement effect and the device designed like a transistor, an instantaneous power density of >10 MW/m^2^ was obtained [113]. Copyright 2021, Springer Nature. (d) Three energy-storage units: (i) flexible lithium-ion batteries [51]. Copyright 2015, WILEY-VCH Verlag GmbH & Co. KGaA, Weinheim; (ii) supercapacitor [78]. Copyright 2020, American Chemical Society; (iii) hybrid supercapacitor [79]. Copyright 2021, Springer Nature. (e) Energy utilization: (i) driving portable electronics [79]. Copyright 2021, Springer Nature; (ii) self-powered sensors [59]. Copyright 2020, The Royal Society of Chemistry; (iii) wireless sensor [80]. Copyright 2016, Springer Nature.

Typical output characteristics of a TENG are alternating current (AC) pulse with high voltage, low current and high internal resistance. Power management is required to achieve AC-to-DC conversion, reduce the voltage, boost the current and obtain impedance matching. Therefore, the self-charging power system (SCPS) is proposed to serve as a sustainable wearable power source. An SCPS consists of four parts, including an energy-harvesting device, energy-management circuit, energy-storage unit and electrical appliances (Fig. 4a). Some of the representative SCPSs are shown in Fig. 4b. Initially, TENG energy harvesting and energy storage were integrated without power utilization optimization. For example, three series-connected fiber-shaped supercapacitors could be directly charged to 5 V by a flexible TENG in 5 s and the storage energy can power a commercial liquid crystal display (Fig. 4bi) [69]. An arch-shaped TENG with an LIB was proposed by Wang *et al.* in which a rectifier was adopted to covert the AC to DC. The LIB can be charged from 0.7 to 2.5 V in 11 h (Fig. 4bii) [70]. Xu *et al.* initially developed an SCPS with a plating stitch technique, which can output the voltage up to 232 V and power density up to 66.13 mW/m^2^. After integrating with a power-management module, the SCPS can constantly power wearable electronics by converting the AC to DC output and improving the energy utilization (Fig. 4biii) [48]. In these systems, the power utilization efficiency is low due to the impedance mismatch between the TENG and energy-storage devices. Suitable conditioning circuits are highly demanded. The output energy of a TENG in one cycle is expressed as the integral of the voltage and the transferred charge in 2D coordinates [3,71]. When the TENG is connected with the electrical appliances, the transferred charge amount *Q_c_* is less than the short-circuit transferred charge amount *Q_sc_.* When a parallel-connected travel switch happens to be short-circuited in a certain motion state, the integral of *V*–*Q* will be further expanded. In an extreme case that the external impedance is infinite (open-circuit state), the integral of *V*–*Q* is the maximum at this moment. Based on the theory, researchers have devoted a lot of effort into designing a series of switching circuits to manage the output of TENGs and improve energy utilization. Xi *et al.* proposed a universal power-management strategy and the matched impedance of TENGs decreased from 35 to 1 MΩ at 80% energy utilization efficiency by the implementation of a DC buck conversion (Fig. 4ci) [72]. Yang *et al.* designed a power-management circuit with an electrostatic vibrator switch [73]. Zhang *et al.* demonstrated a microelectromechanical system (MEMS) plasma switch, which was controlled by the combination of micro-discharge and electrostatic pulling principles [74]. Liu *et al.* demonstrated a switched-capacitor converter using a fractal design with the advantages of high conversion efficiency, minimum output impedance and electrostatic voltage applicability (Fig. 4cii) [75]. Wu *et al.* developed an SCPS with an optimized power-management module, which can constantly power wearable electronics by converting the AC to DC output and improving the energy utilization (Fig. 4ciii) [48]. Detailed summaries on power-management strategies can also be found in recent review papers [76,77].

A high-performance energy-storage unit is also an important part of a self-charging system. Pu *et al.* proposed the LIB belt as a self-charging power unit for future wearable smart electronics; when a textile TENG works in CS mode to charge an LIB belt for 14 h at 0.7 Hz, a discharge capacity of 4.4 mAh/cm at a discharge current of 1 μA has been reached (Fig. 4di) [51]. Han *et al.* developed a highly integrated energy fiber composed of a supercapacitor as an energy-storage unit and a fiber TENG, in which the fiber TENG can sustainable supply electric energy to the supercapacitor (Fig 4dii) [78]. Yin *et al.* also designed a supercapacitor module integrated as the energy-storage unit for the whole system. Compared with the previous integration of an SCPS, this work demonstrated the complementary relationship between two body energy harvesters that scavenge energy from human motion and maximized efficiency and performance (Fig. 4diii) [79]. (Fig. 4d) For demonstrating the real application of a self-charging system, storage electric energy is used to drive (i) portable electronics [79], (ii) self-powered sensors [59] and (iii) wireless sensors (Fig. 4e) [80]. The above TENG structure and power-management strategy designs both provide a clear direction for the future solution of the TENG as a wearable energy source. Table 1 summarizes some of typical TENGs as wearable power sources.

**Table 1.** Representative performance of TENGs as wearable power sources.

Wearable TENG	Output performance	Energy-storage unit	Power management	Application demonstration	References
Flexible all-printed CS-mode TENG	*V_OC_* = 106 V, *I_SC_* = 2.4 μA, 34 mW/m^2^	Capacitor (2 μF, 0–10.2 V in 60 s)	Rectification	Driving electronic watch and thermometer	[54]
Flexible CS-mode TENG	*V_OC_* = 30 V, *I_SC_* = 0.4 μA, 32 mW/m^2^	None	Rectification	Lighting up 234 green commercial LEDs	[47]
Textile-based TENG	*V_OC_* = 232 V, *I_SC_* = 6.3 μA, 66.13 mW/m^2^	Capacitor (15 mF, 0–0.12 V in 300 s)	Direct-current (DC) buck conversion	Driving a pedometer	[48]
Textile-based TENG	*V_OC_* = 25 V, *I_SC_* = 150 nA, 1.008 mW/m^2^	None	Rectification	Lighting up 116 commercial LEDs, function of loading pressure variations	[50]
Textile-based TENG	*V_OC_* = 150 V, *I_SC_* = 4 μA, 393.7 mW/m^2^	Flexible LIB belt (0.4–1.9 V in 4 h)	Rectification	Powering heartbeat meter strap	[51]
Soft tubular TENG	*V_OC_* = 200 V, *I_SC_* = 106 μA, 112.5 mW/m^2^	Capacitor (27 μF, 1.2– 4.2 V in 126 s)	DC buck conversion	Driving portable electronics	[52]
Multi-layer TENG	*V_OC_* = 700 V, *Q_SC_* = 2.2 μC, 7.34 W/m^2^	Capacitor (1 mF, 3.3–3.7 V in 300 s)	Power-management system	Human activity sensors, driving portable electronics	[53]
Stretchable TENG	*V_OC_* = 104 V, *I_SC_* = 8.0 μA, 2.3 W/m^2^	Capacitor (1 μF, 0–8 V in 60 s)	Rectification	Intelligent control for lamp	[55]
Breathable TENG	*V_OC_* = 168 V, *I_SC_* = 8.3 μA, 1.03 W/m^2^	Capacitor (0.47 μF, 0–5 V in 5 s)	Rectification	Lighting up six commercial LEDs	[56]
Stretchable and transparent TENG	*V_OC_* = 255.3 V, *I_SC_* = 22.6 μA, 4.06 mW/m^2^	Capacitor (22 μF, 0–2 V in ≈211 s)	Rectification	Powering electronic watch	[114]
Flexible TENG	*V_OC_* = 70 V, *Jsc* = 30.2 mA/m^2^, 2.79 W/m^2^	Capacitor (2.2 μF, 0–0.5 V in 50 s)	Rectification	Lighting up 80 blue LEDs	[115]
Textile-based DC TENG	*V_OC_* = 90 V, *I_SC_* = 0.45 μA	Supercapacitor (0–1.8 V in 80 s)	None	Driving a calculator and a hygrothermograph	[116]
Textile-based TENG	*V_OC_* = 80 V, *I_SC_* = 13 μA, 824 mW/m^2^	Capacitor (22 μF, 0–880 μC in 700 s)	Rectification	Lighting up 750 LEDs	[117]
Textile-based TENG	*V_OC_* = 1600 V, *I_SC_* = 12 μA, 203 mW/m^2^	Capacitor (47 μF, 0–880 μC in 200 s)	Rectification	Driving a smart watch	[118]
Textile-based TENG	*V_OC_* = 50 V, *I_SC_* = 0.35 μA, 263.36 mW/m^2^	Capacitor (0.68 μF, 0–2.5 V in 7 s)	Rectification	Driving a smart watch	[119]
Textile-based TENG	*V_OC_* = 19 V, *I_SC_* = 0.4 μA, 11 and 0.88 W/m^2^ under compressing state and stretching state	Capacitor (1 μF, 0–3.5 V in 7 s)	Rectification	Active sensors, lighting up 24 LEDs	[120]
Direct-current fabrics TENG	*V_OC_* = 1200 V, *Isc* = 2 μA, 0.75 mW/m^2^	Capacitor (100 μF, 0–4 V in 300 s)	Rectification	Lighting up 99 bulbs and 1053 LEDs	[121]
Textile-based TENG	*V_OC_* = 880 V, *Jsc* = 1.1 μA/cm^2^, 0.14 W/m^2^	Capacitor (16 μF, 0–2 V in 20 s)	Rectification	Lighting up 150 LEDs	[57]

## TENGS AS WEARABLE SELF-POWERED SENSORS

With the booming development of the Internet of Things (IoT) and artificial intelligence technology, billions of sensors are estimated to be connected to the IoT. Such a huge number of sensors will bring a big challenge to traditional power supply technology. In this regard, self-powered sensors based on TENGs will be an optimum option. TENGs could convert mechanical energy to electricity. By analysing the electric signals, the information of the mechanical input could be retrieved. TENG-based self-powered sensors could be utilized for various applications, including physiological signal sensing, biomedical monitoring, human–machine interaction, etc.

### Physiological signal sensing

**Figure 5.**
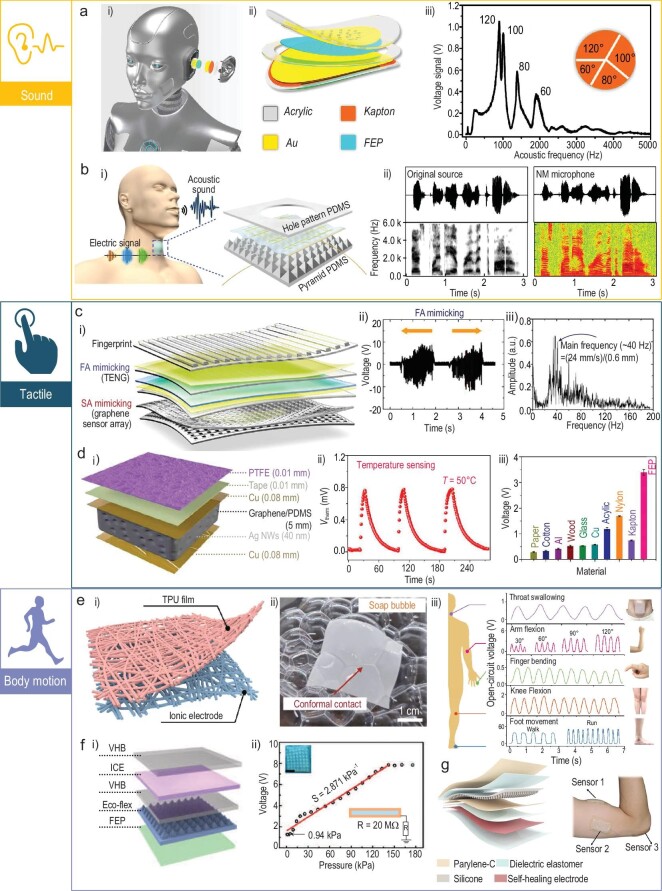
Wearable TENGs for physiological signal sensing applications. (a) A highly sensitive and wide-frequency-range self-powered auditory sensor for both robotics and human hearing aids [81]. Copyright 2018, American Association for the Advancement of Science. (b) A skin attachable microphone [82]. Copyright 2018, American Association for the Advancement of Science. (c) A self-powered tactile sensor for texture recognition [84]. Copyright 2019, American Chemical Society. (d) A multi-functional tactile sensor that could detect pressure, temperature and materials [85]. Copyright 2020, American Association for the Advancement of Science. (e) A breathable ionic mechanoreceptor for body motion sensing [87]. Copyright 2022, Springer Nature Switzerland AG. (f) A stretchable, transparent and thermal ionic sensor for limbs motion sensing [88]. Copyright 2020, WILEY-VCH Verlag GmbH & Co. KGaA. (g) A stretchable, self-healing and skin-mounted sensor for muscle status monitoring [89]. Copyright 2021, American Chemical Society.

In daily life, most physiological activities of human bodies could act as a mechanical stimulus for TENGs. For example, the voice is a specific form of vocal-cord vibrations. Different words could generate distinguished sound waves. TENGs can be utilized to convert these sound waves into electric signals and realize self-powered acoustic sensing (Fig. 5a)and b). Compared with the most commonly used piezoceramics-based commercial auditory sensors, TENG-based sensors could be fabricated to be flexible or stretchable, and thus have great potential in wearable electronics. An inevitable drawback of TENG-based sensors is the low-frequency sensitivity, but this feature also endows them with frequency selectivity. Furthermore, the frequency responsibility of TENG-based auditory sensors could be effectively improved by adjusting the structure of the sensors. A highly sensitive and wide-frequency-range self-powered auditory sensor was therefore developed for both robotics and human hearing aids [81]. This device adopted a layer-by-layer structure and mainly consisted of three parts: a fluorinated ethylene propylene (FEP) film covered with Au as the upper electrode, a spacer and a Kapton membrane covered with an Au lower electrode. During the operation, the Kapton membrane could be free for vibration and the contact separation between the FEP and Kapton film could generate electrical signals. By optimizing the structure of the auditory sensor, the resonant effect could be efficiently inhibited and the sensor could obtain a broad frequency response from 100 to 5000 Hz, which nearly covers the whole range of the human voice. This self-powered auditory sensor could be finally used to control a robot using the human voice and achieve word recognition for recovering hearing-impaired people. Similarly, a skin-attachable microphone also has been reported [82]. Due to the softness of the microphone, it could be attached to the human throat and directly detect the vibration of the vocal cords. The waveform of the electrical signals obtained from the microphone is highly fitted with the original acoustic waveform. Owing to the accurate collection of acoustic signals, a personal voice security system was constructed based on the microphone, which can precisely distinguish the user's voice. To further improve the frequency bandwidth/fidelity and broaden the usage scenario, a wearable water-proof acoustic sensing wristband was developed [83]. The device has a sandwiched structure: polytetrafluoroethylene (PTFE) microparticles filling the gap between two graphite-coated polyethyleneterephthalate (PET) films. When sound waves reached the wristband, the PTFE particles would vibrate under the same frequency. The contact-separation process between the PTFE particles and the graphene will generate electrical signals. This acoustic sensing wristband has a broad frequency response range of 0.1–20 kHz, a stable sensing performance even under sweat conditions and excellent fidelity independent to shapes (whatever is being rolled or flatted).

The contact separation or sliding motion produced by touch movements also could be used as the external mechanical stimuli for TENG sensors. The low-frequency mechanical movement generated by tactile motion could be converted into a large electrical output by TENGs. Therefore, various self-powered tactile sensors could be developed. However, the sensitivity of TENG-based sensors still needs to be improved. By mimicking the function of the skin on fingers, a self-powered flexible tactile texture sensor was proposed (Fig. 5c) [84]. With the aid of this tactile sensor, surface texture recognition could be demonstrated. Through the fast Fourier transformation analysis, the feature of the texture could be effectively extracted from the electrical signals. Subsequently, by means of a deep-learning technique, different textures could be successfully recognized. Then, hierarchically patterned self-powered sensors were further developed that could not only detect pressure and temperature, but recognize different materials (Fig. 5d) [85]. The pressure and temperature detection part of the sensor was composed of a sponge-like PDMS/graphene composite sandwiched by two silver nanowire-coated Cu electrodes. The perception of pressure and temperature could be achieved by the piezo-resistance and pyroelectricity effect of the PDMS/graphene composite. The materials recognition part of the sensor was achieved using a TENG that consisted of the Cu electrode with a layer of PTFE working in single-electrode mode. As the PTFE contacts different materials, the voltage output value of the TENG is different, so the category of the materials could be distinguished. To make the tactile sensor light and breathable, an ultra-thin and stretchable highly sensitive self-powered haptic sensor base on electrospun fibers was developed [86]. The fabricated sensor was stretchable (∼800%), ultra-thin (∼89 μm) and lightweight (∼0.23 g). Due to the features above, this haptic sensor could conformally attach to human skin.

Physical activities of human bodies will produce vast wasted low-frequency mechanical energy, which can be collected for motion sensing by attaching TENGs to relevant positions. Therefore, the softness and stretchability of these devices will have great importance. If the device can be compactly attached to skin, it can capture even the tiny motion of bodies. Wang *et al.* have reported an iontronic TENG for body motion sensing (Fig. 5e) [87]. This mechanoreceptor mainly contains two parts: electrospun thermoplastic polyurethanes (TPU) film and ionic electrode, achieving features like being ultra-thin (∼2.5 μm) and ultra-light (0.076 mg/cm^2^). By attaching the mechanoreceptor to different human body locations, the monitoring of throat swallowing, arm flexion, finger blending, knee flexion and foot movement could be successfully achieved. Also, a stretchable, transparent and thermally stable TENG using solvent-free ionic elastomer electrodes for limbs motion sensing has been proposed (Fig. 5f) [88]. The sensitivity of this TENG-based pressure sensor could reach 2.87 k/Pa. Then, the movement of the wrist, elbow and knee was effectively monitored by attaching the sensor at corresponding positions. Assessment of muscle function has great significance for rehabilitation training and health evaluation. A stretchable, self-healing and skin-mounted active sensor was developed for muscle status monitoring [89]. The triboelectric output, minimal detection limit and response time of this sensor could reach 78.44 V, 0.2 mN and 1.03 ms, respectively. By mounting the active sensors at the positions of the biceps brachii, triceps brachii and elbow, myodynamia and bend angles could be successfully detected.

### Biomedical monitoring

**Figure 6.**
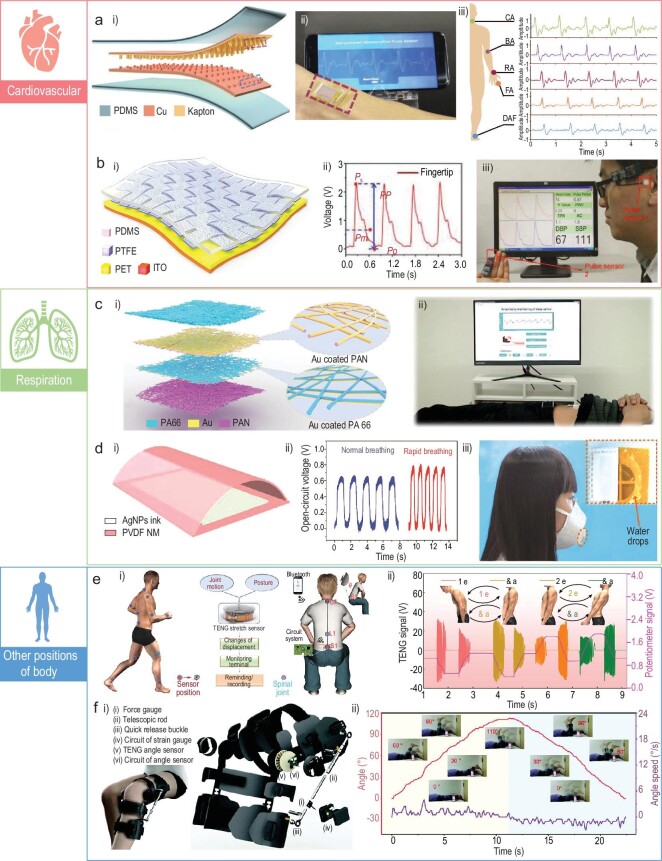
Wearable TENGs for biomedical monitoring. (a) A self-powered pulse sensor for cardiovascular diseases anti-diastole [90]. Copyright 2017, WILEY-VCH Verlag GmbH & Co. KGaA. (b) A woven self-powered pulse and blood pressure sensor [91]. Copyright 2018, WILEY-VCH Verlag GmbH & Co. KGaA. (c) A self-powered e-skin for sleep respiration monitoring [93]. Copyright 2021, Wiley-VCH GmbH. (d) A self-powered mask for respiration monitoring [94]. Copyright 2017, Tsinghua University Press and Springer-Verlag GmbH Germany. (e) A self-powered reel for spine motion monitoring [96]. Copyright 2021, Springer Nature. (f) A self-powered rotation sensing system for self-assessment of knee osteoarthritis patients [97]. Copyright 2022, Wiley-VCH GmbH.

Monitoring various biomedical signals distributed around the body has great significance for early clinical diagnosis or rehabilitation treatment. With the acceleration of population aging, cardiovascular diseases have become one of the greatest threats to human health. Several techniques can now be used to monitor the cardiovascular status of humans. Photoplethysmography (PPG) and electrocardiography (ECG) are the most commonly selected. Though PPG is convenient and could be fabricated into portable devices, the accuracy of test results will be affected by ambient light, distances and skin color. ECG, on the contrary, has good accuracy, but multiple electrodes and clutter wires restrict the further application of ECG devices for wearable electronics. TENGs could effectively collect the low-frequency mechanical disturbances caused by heartbeats and transform them into large electrical output signals for the early diagnosis of cardiovascular diseases. These TENG-based devices usually have a high signal-to-noise ratio and could be easily fabricated to be lightweight, comfortable and flexible. However, the stability of signals is susceptible to other motions of the human body, such as arm swings or walking. Therefore, signal process methods, such as data filters, are beneficial to obtain stable signals. Li's group has reported a self-powered pulse sensor for cardiovascular diseases anti-diastole (Fig. 6a) [90]. The sensor consists of a layer of Cu and Kapton and the whole sensor is encapsulated by PDMS. When the sensor was placed on the wrist, the pulsation of the radial artery would bring contact-separation motions between the Cu and the Kapton. To improve the sensitivity of the sensor, nanowires were fabricated on the surface of the Kapton using an inductively coupled plasma (ICP) process. The signals obtained from the sensor contain abundant information for the anti-diastole of coronary heart disease, atrial septal defects and atrial fibrillation. Apart from the wrist, pulses on the carotid artery, the brachial artery, the radial artery, the finger and the ankle artery area also could be detected using this sensor. Furthermore, a woven structured sensor was proposed that can not only test the pulse, but also measure blood pressure (Fig. 6b) [91]. The sensor could detect the pulses at the wrist, ear, finger and ankle positions. To conduct the measurement of blood pressure, two sensors were placed over the fingertip and ear respectively to detect the pulse wave. By determining the time delay between the two waves, the blood pressure could be calculated. Recently, Ouyang *et al.* developed a bioresorbable pressure sensor for the postoperative care of cardiovascular patients [92]. Materials adopted to construct the sensor are all biodegradable and the sensor is implantable. Polylactic acid (PLA) was chosen for encapsulation and one of the triboelectrification materials. Mg was selected for the electrodes and other triboelectrification materials. For correct operation, the sensor should be rolled on the blood vessel. When vascular occlusion occurs, an abnormal electrical signal will be generated by the sensor. Besides vascular occlusion, the pressure sensor also could be used to identify dyspnea and arrhythmia.

Respiration is an uninterrupted vital biomechanical behavior that also contains abundant information related to health status. The respiration process always brings a low-frequency movement of the chest or belly. Therefore, TENG-based sensors are generally put on these locations to monitor the intensity, depth and rate of respiration. In this condition, a self-powered e-skin was fabricated to detect the movement of the abdomen caused by respiration, as shown in Fig. 6c)[93]. During the inhaled breath processing, the abdomen was pushing out, making the electrification materials of the TENG contact. On the contrary, during the exhaled breath processing, the electrification materials were separated. Through a data process program, the frequency and amplitude of respiration could be tested. After that, a sleep monitoring system was constructed and the obstructive sleep apnea-hypopnea syndrome could be effectively detected. Alternatively, the airflow out from the mouth or nose also contains abundant mechanical energy. Sensors could be integrated with a facial mask to sense the respiration status, as illustrated in Fig. 6d)[94]. The triboelectrification layer of the TENG is an electrospinning poly(vinylidene fluoride) (PVDF) nano-membrane and the electrode is screen-printed Ag nanoparticles of ink. The airflow coming from the mouth will cause the contact-separation movement between the PVDF and the electrode. The output generated from the TENG could be used to monitor the respiration status of humans. One main function of respiration is to exchange gas between organisms and the atmosphere. Therefore, the expiratory gas could be an indicator of health status. TENG-based self-powered sensors also could be used to detect the chemicals of the gas. Wang *et al.* have reported a facial respiration-driven gas sensor [95]. The triboelectrification layer of the TENG in this work is Ce-doped ZnO-polyaniline (PANI). The NH_3_ in the gas will react with the Ce-doped ZnO-PANI and decrease the output of the TENG. Therefore, this sensor could be used to diagnose kidney-relevant diseases.

The monitoring of human motion could be a reference for orthopedic/neural disease diagnosis and rehabilitation assessment. Therefore, a retractable and wearable badge reel was developed for monitoring human spine motions (Fig. 6e) [96]. The reel adopts the annulus structure and the key component of it is a circular sliding TENG. During the operation process, the reel was fixed at the position of the spine tail and the rope connected with the rotor of the reel was fixed on the back of the neck. When the spine was bent, the rope would drive the rotor to spin and generate sine-wave-like electric signals. According to the frequency and peak numbers, the speed and angles of the spine bend motion could be detected. These data can be utilized to prevent spinal disorders for those long-term sitting workers or help to assess recovery status after injuries. Knee osteoarthritis has been considered to be one of the most common age-associated diseases. This disease could be easily treated, but due to limited medical resources in hospitals, patients are suffering during the rehabilitation process. In this case, a portable, modular and self-powered rotation sensing system was developed for the self-assessment of knee osteoarthritis patients during the recovery process (Fig. 6f) [97]. Relying on the series of the mechanical exoskeleton, a rotation TENG was fixed on the side of the knees. Aiding the TENG with a force gauge, the speed, angle and moment of force could be effectively measured. Then, facilitated by an application program and deep-learning technique, patients’ daily activities could be assessed and home training suggestions would be given out. Lumbar degenerative disease is another disease that disturbs aged people and is usually carried with neurogenic intermittent claudication or lower limb pain. Traditional diagnosis of this disease often involves magnetic response imaging, which has an uncertain clinical correlation. A self-powered active-matrix sensing array was fabricated for plantar pressure measurement during walking to improve the diagnosis of lumbar degenerative disease [98]. The sensor matrix was integrated into the shoe pad and, with the aid of a supervised learning algorithm, the matrix can recognize half-squat, squat, jump, walk and jog with an accuracy of ≤99.2%. Furthermore, after testing 62 clinical samples of lumbar degenerative patients, the artificial intelligence diagnosis accuracy could reach 100%.

Meanwhile, there are also some challenges in biocompatible TENGs for wearable self-powered sensors. First, the skin affinity and breathability of the device should be synthetically considered to obtain both excellent performance and comfort. Materials that have both skin affinity and breathability are highly in demand. For those implantable TENGs, a balance between stability and biodegradability of the device should be taken into account so that the operation and degradation process can be precisely controlled. Therefore, new strategies to design controllable and biodegradable materials should be exploited.

### Human–machine interaction (HMI)

**Figure 7.**
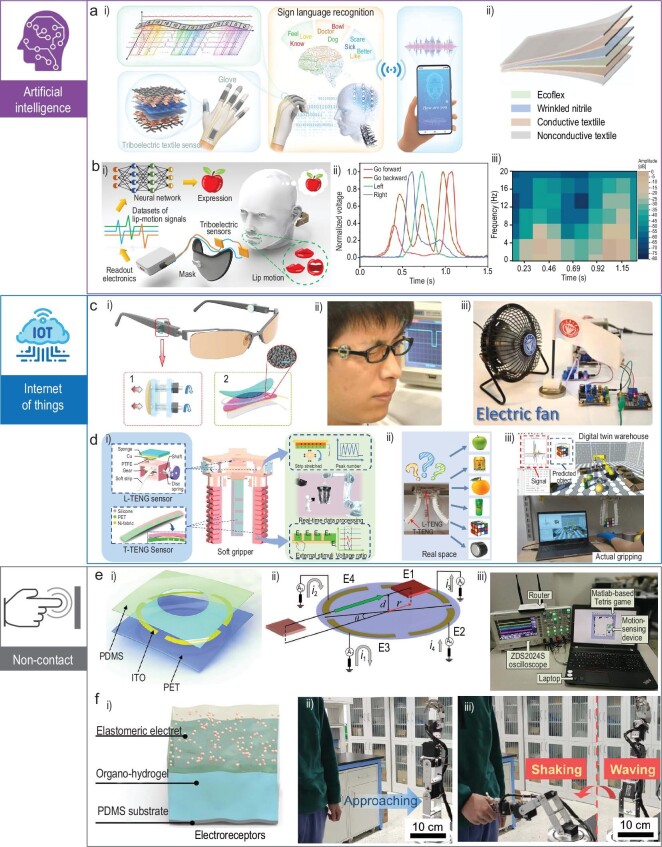
Wearable TENGs for human–machine interaction. (a) A sign-to-speech translation sensor for gesture recognition [99]. Copyright 2021, Springer Nature. (b) A self-powered facial lip-language decoding system [101]. Copyright 2022, Springer Nature. (c) A eye-triggered self-powered sensor that could control multiple electronics [102]. Copyright 2017, American Association for the Advancement of Science. (d) Triboelectric nanogenerator sensors for soft grippers [103]. Copyright 2020, Springer Nature. (e) A self-powered non-contact sensor for motion sensing [105]. Copyright 2017, WILEY-VCH Verlag GmbH & Co. KGaA. (f) An artificial electroreceptor for precontact somatosensation [109]. Copyright 2022, American Association for the Advancement of Science.

HMI systems with wearable and flexible sensors are an inevitable future tendency. Wearable devices enable the HMI system to acquire real-time human physiology or surrounding signals. Combined with the artificial intelligence technique, self-powered TENG sensors could exhibit wide applications for the construction of intelligent life. Zhou *et al.* have developed a sign-to-speech translation sensor array assisted by machine learning [99]. Fiber-shaped TENG-based sensors were placed on each of the fingers and hand-gesture movements could be converted by the sensor arrays into electrical signals. The generated voltage signals are classified by the machine-learning algorithm to improve the accuracy of the translation. Finally, the gestures could be translated into words and displayed on a mobile phone, eliminating the communication barrier between signers and non-signers. Similarly, a triboelectric smart glove has also been reported for sign-language recognition and virtual reality (VR) communication, as shown in Fig. 7a)[100]. The TENG-based sensors were distributed on each joint of the hand and the bend or release motion of the fingers will all generate electrical signals. The different combinations of signals represent different words. By wearing this glove, the human could successfully communicate with a virtual character in the VR scenario. Except for hand-gesture sign language, lip language also could be sensed using TENG-based sensors, as illustrated in Fig. 7b)[101]. For vocal-cord lesions, laryngeal or lingual injuries, lip language is an effective way to communicate in daily life and without occupying the hands. However, it is difficult to understand lip languages for people without professional training. A lip-language decoding system based on triboelectric sensors thus was developed. The sensor was integrated with a mask and detected lip languages by sensing lip motions. Aided by a well-trained dilated recurrent neural network model, the system could reach a test accuracy of 94.5% in training 20 classes with 100 samples each.

With the appearance of 5G technology, the era of the IoT is coming. TENGs also could be used as sensors for the construction of the IoT. Pu *et al.* have developed a self-powered eye-motion-triggered sensor for mechnosensational communication systems (Fig. 7c) [102]. The TENGs-based sensor adopted a contact-separation structure, with latex and FEP as the triboelectrification layers. This self-powered sensor was installed on the framework of a pair of glass. When blinking the eyes, natural latex will contact the FEP and generate a voltage signal of ≤750 mV. By integrating with a Microprogrammed Control Unit, the self-powered eye-motion sensor could be used to control several devices, including table lamps, electric fans, bells, etc. Various grippers have been widely used for assembling, sorting and carrying, etc. Developing hand-like grippers that possess flexibility and perception will have great significance in the industrial field. TENG sensors have been reported for soft grippers, as shown in Fig. 7d)[103]. A rotation TENG was installed at the joint position of the gripper for angle sensing and a sliding model TENG was installed on the arm of the gripper for the detection of sliding speed, contact position and gripping mode. After that, the soft gripper is successfully demonstrated to perceive the gripping status and realize object identification by leveraging machine-learning technology. In the virtual-reality environment, the soft gripper has also shown great potential for digital twin applications, e.g. production management and prediction of situations in smart factories. Hu's group fabricated a triboelectric quantization sensor for the gesture control of a robot arm [104]. This TENG will only generate positive or negative signals when the slider moves in a single direction. For example, as the sensor was installed on the fingers, the positive/negative voltage pulses represent the flexion/extension of the finger. Furthermore, by counting the number of generated pulses, the angular position of the fingers also could be determined. Finally, a robot arm could be precisely manipulated using the sensor and the minimum resolution is only 3.8°.

As introduced before, the outputs of TENGs are generated by the coupling of the triboelectrification effect and electrostatic induction effect. By only utilizing the electrostatic induction effect, TENGs could serve as various non-contact sensors. Compared with other types of non-contact sensors, such as ultrasound transducers, light-sensing elements and electromagnetic technologies, these electrostatic sensors will not be affected by light conditions, temperatures or weather variations. In addition, flexibility and stretchability are also unique advantages of TENG-based non-contact sensors. However, the output signals will be liable to be affected by moisture. Efforts still need to be made in overcoming this disadvantage. Wu *et al.* have reported a self-powered non-contact electronic skin for motion sensing, as demonstrated in Fig. 7e)[105]. The electronic skin consists of an indium tin oxide (ITO)-coated PET and a layer of PDMS. The four planar ITO electrodes are distributed in a circle. When an object carrying charges approaches, the four electrodes will all generate a voltage signal and the ratios of the peak voltages are used to determine the displacement of the object. Similarly, a self-powered electronic skin array also was developed for non-contact displacement sensing [106]. The substrate of the array was selected for the PDMS and patterned silver nanowires were spray-coated onto the PDMS as the electrodes. For displacement sensing, a corona charge PTFE was pasted onto the finger, acting as the signal resource. The displacement of the finger was calculated in the rectangular coordinate system with three degrees of freedom according to the obtained voltage signals from the electrodes. The resolution of the array could reach up to 0.75 mm, 1.07 mm, 2.20°. To further improve the functionality of self-powered sensors, the natural world should always be a source of inspiration. In deep seas, some elasmobranch fish, such as sharks or skates, use electroreceptors distributed on the skin to detect prey through the electric field. Inspired by these creatures, an artificial electroreceptor was fabricated for precontact somatosensation, as shown in Fig. 7f)[107]. To endow the electroreceptor with excellent sensitivity and stretchability, the electrode was selected as an organo-hydrogel and the dielectric layer was chosen as the elastomeric electret. The electroreceptor could perceive the approach of not only insulators, but also metals. The matrix of this electroreceptor together with machine-learning algorithms demonstrates the feasibility of non-contact 3D surface profile recognition.

During the fabrication process of wearable TENGs that are utilized in human–machine interfaces, the following factors should be considered. First, sufficient flexibility and stretchability of TENGs are required for joint motion sensing or body positions that will undergo substantial deformations. Second, a good signal-to-noise ratio of TENGs is necessary to ensure the success of the HMI even under a tiny motion. Third, proper encapsulation is needed to prevent the TENG from the influence of the environment.

## SUMMARIES AND PROSPECTS

In summary, significant progress has been made in wearable TENGs as power sources and as self-powered sensors. As power sources, the TENG is promising for achieving the energy autonomy of wearable electronics, i.e. reducing or even eliminating battery recharging/replacing maintenance. This is an ideal goal and at least is possible for many low-power-consumption electronics. The abovementioned progress has shown that the performance of TENGs can be greatly improved using several approaches and, in the meantime, functionalities have been enriched through materials/structures engineering. TENGs in different forms can be designed for harvesting mechanical energies of nearly any moving part of the human body. As self-powered sensors, a series of innovative devices have been constructed to detect the sound, tactile, joint/muscle motion and physiological signals of human bodies, with demonstrated applications in the IoT, human–machine interfaces and health/medical monitoring. Despite this progress, the following aspects may still require future efforts.

For wearable power sources, the following challenges need to be addressed. (i) The output power performances are of prime importance. Recently, several strategies have been found to be very efficient. The excitation of a TENG, using an extra TENG or through a self-excitation circuit [62], has been proven to be highly efficient in boosting surface charges and device output power. Future research may consider implementing these strategies in wearable/flexible/stretchable devices. Furthermore, materials engineering seems to be a facile approach to improving the power, such as tuning the surface functional groups or morphologies, pre-charging the surface with static charges and tuning the dielectric properties or charge trapping properties using inorganic fillers [108]. The state-of-the-art areal surface charge density can reach ∼8.8 mC/m^2^ and the average power can reach the mW scale [109]. Nevertheless, it may be challenging to implement many of these strategies in wearable devices with the specifically required light weight, comfort, breathability or washability. (ii) The influences of environmental conditions should be lowered, such as humidity and sweat. One advantage of the TENG is that it is relatively insensitive to temperature and sunlight, but it can be degraded at high humidity, which accelerates the surface charge dissipation and lowers the output power. This can be facilely solved by appropriate packaging, but high power performance, efficient packaging and wearing comfort should be satisfied simultaneously. For example, a spun hollow fiber is shown to operate normally even underwater [110]. More investigation into this aspect is still highly demanded. (iii) Systematic studies on self-charging power systems are urgently required from the engineering perspective. In most cases, the TENG has to be integrated together with a conditioning circuit to provide constant and stable electrical power to the wearable electronics. In this circuit, an energy-storage unit of either capacitors or batteries is demanded and their capacity should be designed based on the power consumption of the electronics and/or the output power of the TENG. The energy-transfer efficiency has been optimized according to previous reports [111]. Further studies on optimizing the efficiency, standardizing the circuit and miniaturizing the circuit size are highly demanded.

As for wearable self-powered sensors, the following challenges are required to be addressed. (i) The sensing performances should be further optimized. The critical sensing parameters might vary with applications. The advantage is that the TENG sensor could optimize the sensitivity, linearity, detecting limit or response time through facile structures/materials designs, but the sensing performances have to be outstanding over or comparable with competing technologies. Killer applications should be found and targeted. (ii) Scalable fabrication has always been a challenge for flexible sensors, not only for TENG-based sensors. The repeatability and stability of sensors are prerequisites for commercial applications, which, however, will be an issue especially when flexible, stretchable soft materials are used. This is typically because of the viscoelasticity and low elastic resilience of these materials. Therefore, there is a dilemma between multi-functionalities and sensing stabilities in many cases, which may require compromises according to the specific applications. (iii) There is always a pursuit for better ‘intelligence’ of wearable electronics. Machine-learning technology has brought revolutions to many aspects of our daily lives. Considering the versatile capabilities of TENG sensors in detecting a variety of human-related signals, the combination of these sensing capabilities with artificial intelligence are expected to change lifestyles in healthcare, communication and recreation.

**Figure 8.**
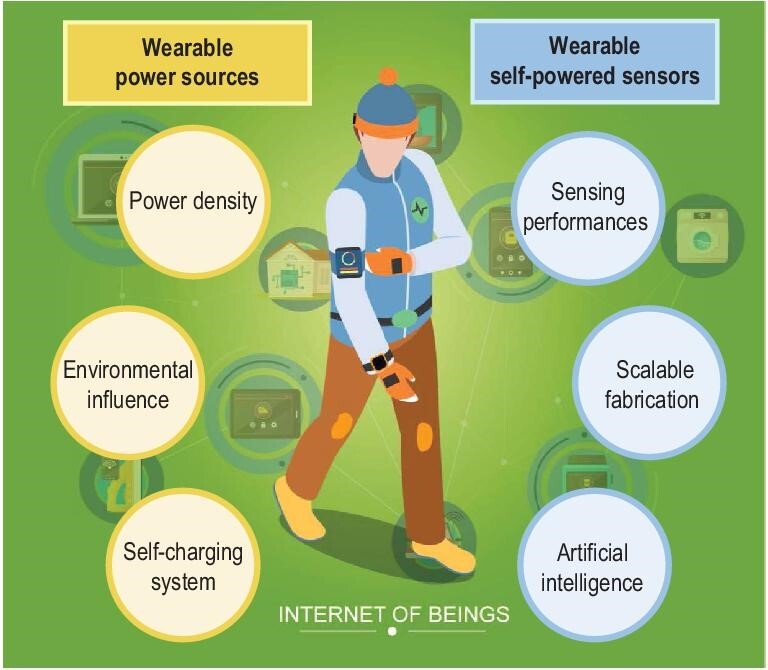
Prospects for the wearable TENGs as power sources and self-powered sensors.

With the development of smart wearable technologies, we are now augmented beyond our biological capabilities in communication, healthcare and so on. Other than the IoT, the internet of bodies or internet beings is expected to advance rapidly and further reform our lifestyles, as shown in Fig. 8. Energy supply and information acquisition are the basics for achieving these goals. Therefore, considering the potential dual function of the TENG, it is expected to contribute crucially to these technology advancements and more efforts are required to promote its progress.
